# Dual-drug loaded nanoparticles of Epigallocatechin-3-gallate (EGCG)/Ascorbic acid enhance therapeutic efficacy of EGCG in a APPswe/PS1dE9 Alzheimer's disease mice model

**DOI:** 10.1016/j.jconrel.2019.03.010

**Published:** 2019-05-10

**Authors:** Amanda Cano, Miren Ettcheto, Jui-Hsien Chang, Emma Barroso, Marta Espina, Britta A. Kühne, Marta Barenys, Carmen Auladell, Jaume Folch, Eliana B. Souto, Antoni Camins, Patric Turowski, Maria Luisa García

**Affiliations:** a Department of Pharmacy, Pharmaceutical Technology and Physical Chemistry, Faculty of Pharmacy and Food Sciences, University of Barcelona, Spain; b Institute of Nanoscience and Nanotechnology (IN2UB), Barcelona, Spain; c Biomedical Research Networking Centre in Neurodegenerative Diseases (CIBERNED), Madrid, Spain; d UCL Institute of Ophthalmology, University College of London, United Kingdom.; e Department of Pharmacology, Toxicology and Therapeutic Chemistry, Faculty of Pharmacy and Food Sciences, University of Barcelona, Spain; f Unit of Biochemistry and Pharmacology, Faculty of Medicine and Health Sciences, University of Rovira i Virgili, Reus, Tarragona, Spain; g Biomedical Research Center in Diabetes and Associated Metabolic Diseases (CIBERDEM)-Health Institute Carlos III, Barcelona, Spain; h Research Institute-Hospital Sant Joan de Déu, Esplugues de Llobregat, Barcelona, Spain; i Department of Cellular Biology, Physiology and Immunology, Faculty of Biology, University of Barcelona, Spain; j Department of Pharmaceutical Technology, Faculty of Pharmacy, University of Coimbra, Coimbra, Portugal; k REQUIMTE/LAQV, Group of Pharmaceutical Technology, Faculty of Pharmacy, University of Coimbra, Coimbra, Portugal

**Keywords:** Epigallocatechin gallate, EGCG, Polymeric nanoparticles, PLGA-PEG, Alzheimer's disease, APP/PS1 mice, AA, ascorbic acid, Aβ, amyloid-β, APP/PS1, APPswe/PS1dE9, BBB, blood-brain barrier, BMVECs, brain microvascular endothelial cells, EE, encapsulation efficiency, EGCG, epigallocatechin-3-gallate, EGCG/AA NPs, dual-drug loading PEGylated PLGA nanoparticles of EGCG and AA, EGCG/AA NPs-Rho, EGCG/AA NPs covalently labelled with Rhodamine 110, FITC-dextran, Fluorescein isothiocyanate-dextran, FTIR, Fourier transform infrared spectroscopy, GFAP, glial fibrillary acidic protein, HPLC, high performance liquid chromatography, IL-6, interleukin 6, iNOS, inducible nitric oxide synthase, i.p., intraperitoneal, MWM, Morris Water Maze, NOR, Novel Object Recognition, NPs, nanoparticles, PDI, polydispersity index, PEG, Polyethylene glycol, PFA, paraformaldehyde, PLGA, poly(lactic-*co*-glycolic acid), Rho, Rhodamine 110, SYN, Synaptophysin, TEER, transendothelial electric resistance, ThS, Thioflavin-S, TNFα, tumor necrosis factor α, WT, wild-type, XRD, X ray diffraction, Z_av_, average particle size, ZP, zeta potential

## Abstract

Epigallocatechin-3-gallate (EGCG) is a candidate for treatment of Alzheimer's disease (AD) but its inherent instability limits bioavailability and effectiveness. We found that EGCG displayed increased stability when formulated as dual-drug loaded PEGylated PLGA nanoparticles (EGCG/AA NPs). Oral administration of EGCG/AA NPs in mice resulted in EGCG accumulation in all major organs, including the brain. Pharmacokinetic comparison of plasma and brain accumulation following oral administration of free or EGCG/AA NPs showed that, whilst in both cases initial EGCG concentrations were similar, long-term (5–25 h) concentrations were ca. 5 fold higher with EGCG/AA NPs. No evidence was found that EGCG/AA NPs utilised a specific pathway across the blood-brain barrier (BBB). However, EGCG, empty NPs and EGCG/AA NPs all induced tight junction disruption and opened the BBB in vitro and ex vivo. Oral treatment of APPswe/PS1dE9 (APP/PS1) mice, a familial model of AD, with EGCG/AA NPs resulted in a marked increase in synapses, as judged by synaptophysin (SYP) expression, and reduction of neuroinflammation as well as amyloid β (Aβ) plaque burden and cortical levels of soluble and insoluble Aβ_(1-42)_ peptide. These morphological changes were accompanied by significantly enhanced spatial learning and memory. Mechanistically, we propose that stabilisation of EGCG in NPs complexes and a destabilized BBB led to higher therapeutic EGCG concentrations in the brain. Thus EGCG/AA NPs have the potential to be developed as a safe and strategy for the treatment of AD.

## Graphical abstract

**Figure:**
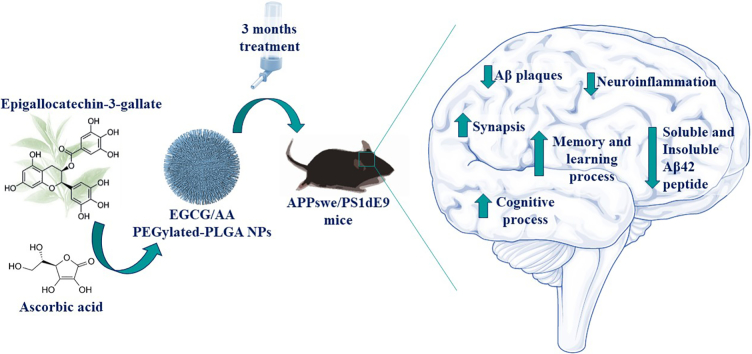

## 1 Background

AD is the most common form of dementia worldwide [1] and involves 60–80% of all reported dementias. It is characterized by cognitive decline and, ultimately, by an incapacitation of basic functions, such as swallowing or walking that leads to death [2]. In 2005, the *Delphi Consensus Study* predicted that, by the year 2020, 42.3 million people in the world will have developed dementia, and that by 2040 this number will have doubled [3]. This increase in dementia cases will be accompanied by a large economic burden, which currently stands at around a trillion dollar worldwide [4].

The incidence of AD increases with age, but its exact etiology remains unknown. The main hypothesis of pathogenesis focuses on the alteration in the amyloidogenic pathway and Tau hyperphosphorylation. This is based on observations of increased accumulation of Aβ plaques and induction of formation of neurofibrillary tangles in the brain, respectively, both of which can lead to neuronal death and subsequent dementia [5]. In the last decade, it has been described that alterations in both pathways are closely related to the onset of the disease, but also that other physiological alterations such the deregulation of cholesterol homeostasis, insulin signaling, neuroinflammatory processes, mitochondrial alterations or oxidative stress contribute to the pathogenesis of this multifactorial disorder [6,7].

Due to its unknown etiology and the late appearance of the first symptoms (which are detected when neurodegeneration is already established), early diagnosis and effective treatment are very challenging [8,9]. Currently, the only drugs approved for AD treatment are the acetylcholinesterase inhibitors donepezil, galantamine and rivastigmine, and the NMDA receptor antagonist memantine, for mild to moderate and moderate to severe stages, respectively [1,5]. However, since none of these drugs stop the progression of the disease, novel therapeutic approaches for the treatment of AD are urgently needed [5].

A number of new pharmacological strategies for AD have been described [[10], [11], [12]]. One of them focuses on EGCG, the most abundant polyphenol of green tea plant *Camellia sinensis* [13]. This catechin has a strong chelating and antioxidant activity, due to two gallocatechol rings which can directly remove free radicals with high efficacy [14].

EGCG has demonstrated therapeutic effectiveness in several diseases, such as Down's syndrome, cancer and a variety of neurological disorders [[15], [16], [17]] and is considered a safe molecule [18]. Some cases of liver damage with EGCG consumptions higher than 800 mg/day have been reported, but in most cases these are attributed to idiosyncratic reactions [18].

With regard to AD, pre-clinical and clinical trials have shown that EGCG can act at different steps, such inhibiting TAU aggregation, reducing Aβ accumulation, suppressing tumor necrosis factor α (TNFα), inducible nitric oxide synthase (iNOS) or interleukin 6 (IL-6) expression, or even protecting mitochondrial function [[19], [20], [21], [22]]. However, EGCG possesses many pharmacochemical disadvantages, in particular high instability, resulting in low EGCG bioavailability and, consequently, its effectiveness [23,24]. EGCG degradation is primarily caused by oxidation of the polyphenolic structure. Thus, potent antioxidant molecules, such ascorbic acid (AA), are frequently added to reverse this effect. In addition to scavenging oxygen and protecting double bonds, AA also adds anti-inflammatory properties to resulting formulations [25,26].

Another strategy to reduce degradation of drugs is their incorporation into nanostructured systems. In recent years, the use of nanotechnology as a therapeutic strategy for targeting and drug delivery has been widely explored for diseases such as cancer, diabetes or neurodegenerative disorders [[27], [28], [29]].

Among several types of nanocarriers, polymeric NPs have been widely exploited for drug delivery and targeting, due to their versatility and production facilities. These nanosystems display a high loading capacity of a range of chemically different drugs. In addition, they offer the possibility of attaching molecules to their surface for targeting, therefore representing an optimal alternative for the administration of drugs [30,31]. Due to its biocompatibility, biodegradability and non-toxicity, poly (lactic-*co*-glycolic acid) (PLGA) is one of the most commonly used polymers for the preparation of NPs [32]. Additionally, surface PEGylation of polymeric NPs is often used to enhance in vivo half-life. Surface tailoring NPs with polyethylene glycol (PEG) chains reduces aggregation, improves aqueous solubility and minimizes opsonisation, resulting in demonstrably lower immunogenicity and enhanced long term stability of the nanosystem [33,34].

We hypothesized that loading of EGCG in PEGylated PLGA NPs will improve physicochemical stability of the molecule and, consequently, its bioavailability and therapeutic efficacy. Thus, the aims of this work were to develop and characterize PEGylated PLGA NPs of EGCG under an AA antioxidant environment and to evaluate their effectiveness in a APP/PS1 double transgenic mouse model.

## 2 Materials and methods

### 2.1 Preparation of EGCG/AA NPs

EGCG/AA NPs were prepared by the double emulsion method [35] in three steps (Supplementary Fig. S1). (i) EGCG (Capotchem Hangzhou, P.R.China) and AA (Sigma Aldrich, Madrid, Spain) were dissolved in 1 ml of an aqueous phase (W_1_) at pH 3.5 and emulsified with 1.5 ml of an oil phase (O) composed of ethyl acetate containing the dissolved polymeric matrix (PLGA with a 5% of PEG content) (Evonik Co., Birmingham, USA). A simple emulsion (W_1_/O) was prepared by ultrasound using an ultrasounds probe (Sonics&Materials, INC. Newtown, USA) at 37% amplitude during 30 s. (ii) 2 ml of Tween®80 2.5% (pH 4.5) (W_2_) (Sigma Aldrich, Madrid, Spain) was added to W_1_/O and the mixture again subjected to ultrasound (37% amplitude) for 3 min, to yield a double emulsion (W_1_/O/W_2_). The entire procedure was carried out on ice. (iii) 2 ml of Tween®80 0.04% was added dropwise to stabilize the emulsion. Finally, the organic solvent was evaporated by stirring for 24 h, resulting in a final volume of 5 ml.

### 2.2 Optimization

Final formulation of EGCG/AA NPs with optimal physicochemical characteristics was found using a 2^3^ central composite factorial design allowing optimisation with a minimum number of experiments providing maximum information. A total of 16 experiments were run as described above in triplicate, with varying concentrations of EGCG, AA and polymer (independent variables). The surfactant concentration was left fixed, intended for chronic administration, in order to increase at maximum nanocarrier stability and enhance tissue penetration [36]. The dependent variables included average particle size (Z_av_), polydispersity index (PDI), zeta potential (ZP) and entrapment efficiency (EE). The predicted response Y can be obtained from the non-liner quadratic model equation as follows Eq. (1),

(1) $$Y=\beta_{0}+\beta_{1}X_{1}+\beta_{2}X_{2}+\beta_{3}X_{3}+\beta_{11}X_{1}^{2}+\beta_{22}X_{2}^{2}+\beta_{33}X_{3}^{2}+\beta_{12}X_{1}X_{2}+\beta_{13}X_{1}X_{3}+\beta_{23}X_{2}X_{3}$$

where Y is the measured response, β_0_ the intercept, β_1_ to β_3_ the linear coefficients, β_11,_ β_22_ and β_33_ the square coefficients, β_12,_ β_23_ and β_23_ the interaction coefficients and X_1_, X_2_ and X_3_ are the independent studied factors. Statgraphics Plus (16.1.18) was used to compute these models and the effect of the factors was statically evaluated by analysis of variance (ANOVA).

### 2.3 In vitro drug release

The release profile of EGCG from the polymeric matrix was determined by bulk-equilibrium direct dialysis as described by Fangueiro et al., with some modifications [37]. Briefly, 10 ml of EGCG/AA NPs were placed in a dialysis bag (Medicell International Ltd. MWCO 12–14,000) and dialysed against 140 ml of release medium, composed of EtOH (25%, v/v), Transcutol®P (5%, v/v) and AA (0.25% w/v), pH 3 (to ensure compound stability and mimic the acid environment of the stomach). 1 ml samples were withdrawn at regular time intervals during 24 h from the release medium and EGCG content analysed by HPLC as described elsewhere [38]. Total volume of release medium was kept constant by replacement with fresh buffer throughout the experiment.

### 2.4 Stability study

Stability of drug and NPs was carried out for: 1) the formulation of EGCG-AA NPs; 2) EGCG in water; 3) EGCG in an aqueous solution of AA 0.25%; and 4) EGCG within the NPs formulation at storage temperatures of 4 and 25 °C.

The stability study of NPs was performed with the optical analyser Turbiscan®Lab (Formulation, L'Union, France) as described elsewhere [39]. Samples were scanned every hour for a period of 24 h. This process was repeated every month. Z_av_, PDI and ZP of were also measured monthly.

Drug content of the NPs over time was measured at predefined times. 300 μl of acetonitrile was added to 50 μl of EGCG/AA NPs to break the polymer structure. Then, the total amount of EGCG was determined by HPLC [38]. EGCG in water and AA 0.25% solution was also assessed by direct HPLC measurement. EGCG concentrations were derived by extrapolation to a standard curve of EGCG at 25–500 μg/ml.

### 2.5 Interaction studies

EGCG/AA NPs were ultracentrifuged (Optima® LE-BOK Ultracentrifuge, Beckman, USA) at 25,000 g, 15 °C, for 15 min. Resulting pellets were dried and pulverized.

### 2.6 FTIR

In order to evaluate potentially new bonds generated in the manufacturing process of EGCG/AA NPs a Fourier transform infrared (FTIR) study of different components was carried out. For this, a Nicolet iZ10 with an ATR diamond and DTGS detector (Thermo Scientific) was used. 3 mg of powdered sample was added to 100 mg of KBr and scanned at 500–4000 cm^−1^ in an inert nitrogen/argon atmosphere.

### 2.7 Differential scanning calorimetry

Differential scanning calorimetry (DSC) was carried out as previously described [40]. Briefly, samples were weighed (1–3 mg) in a perforated aluminium pan and analysed in a TA 4000 system (Mettler, Greifensee, Switzerland) equipped with a DSC 25 cell. Heating was performed under a nitrogen atmosphere and a flow rate of 10 °C/min (40–280 °C). Thermograms were analysed with STARe V 9.01 dB software (Mettler, Greifensee, Switzerland).

### 2.8 Nanoparticles transport across the BBB in vitro

Primary brain microvascular endothelial cells (BMVECs) were isolated from rat cortical grey matter microvessels as described elsewhere [41] and seeded onto collagen IV/fibronectin coated Transwells® (12 mm, Costar 3401, Sigma Aldrich, UK) at high density (6 rat brains per 50 cm^2^). BMVECs were cultured in EBM®-2 MV (Lonza, UK) supplemented with 5 μg/ml puromycin during the initial 3 days, until transendothelial electric resistance (TEER) was above 200 Ω/cm^2^ (after 2–3 weeks).

In order to measure transport across this in vitro BBB, EGCG/AA NPs were covalently labelled with Rhodamine 110 (Sigma Aldrich, Madrid, Spain) (12 mg/g of labelled polymer) as previously described [42,43] to produce EGCG/AA NPs-Rho. These were then added at increasing concentrations to the apical side of primary rat BMVECs grown on Transwell® filters. 50 μl of medium were removed from the basal chamber (and replaced by fresh medium) at 20 min intervals for 24 h. Fluorescence was measured in a Safire microplate reader (Tecan, Reading, UK) at 496/520 nm.

### 2.9 Effect of EGCG/AA NPs on the in vitro BBB

Flux of 4 kDa fluorescein (FITC dextran) (Sigma Aldrich, UK) across primary rat BMVECs was measured as described previously [41]. Baseline flux was recorded for 2 h at which point free or encapsulated EGCG was added to the apical chamber and flux recorded for another 2–3 h. Changes of flux were expressed as linear changes of flux before and after addition of EGCG or NPs samples.

Alternatively, primary rat BMVECs were seeded on collagen type IV/fibronectin gold-coated ECIS 8W10E electrode arrays (Applied Biophysics) and real-time impedance changes and extrapolated TEER measured in response to EGCG and NPs treatments [41].

### 2.10 Rat BMVECs staining

Monolayer of rat primary BMVECs grown on collagen IV/fibronectin coated Transwell® filters were treated with free or encapsulated EGCG. After 2 h, cells were fixed with −20 °C MeOH and then stained for Cldn-5 (1200, Thermo Fisher Scientific, UK) and nuclear DNA (Bisbenzimide) exactly as previously described [41]. Images were acquired on a Zeiss Axioskop 2 (Cambridge, UK) using a Hamamatsu camera and HCimage software (Hamamatsu Photonics, Hertfordshire, UK).

### 2.11 Effect of EGCG/AA NPs on the neurovascular unit ex vivo

Rats were asphyxiated using CO_2_ and immediately processed for bilateral carotid arteries cannulation. The head was first perfused with heparin (300 U/ml in isotonic saline) and then with cardioplegic solution (10 mM MgCl_2_, 110 mM NaCl, 8 mM KCl, 10 mM HEPES, 1 mM CaCl_2_, 10 μM isoproterenol), known to protect blood-neural barriers [44]. Perfusates containing Evans Blue (5 g/l in 10% BSA) in cardioplegic solution with or without EGCG or NPs at various concentrations were administered simultaneously via both carotid cannulae under equal delivery pressure (thus avoiding mixing at the Circle of Willis). After 1 h incubation, the drug-containing perfusates were washed out and brains were fixed with 4% PFA (*w*/*v* in PBS) for 24 h before sectioning and imaging of Evans Blue accumulation in brain areas on an Olympus SZX16 stereomicroscope.

### 2.12 In vivo studies

6 months-old male APP/PS1 and control wild type C57BL/6 (WT) mice were used [45]. These transgenic mice express a Swedish (K594M/N595L) mutation of a chimeric mouse/human APP (mo/huAPP695swe), together with the human exon-9-deleted variant of PS1 (PS1-dE9), all of which leads to high expression of Aβ peptide. Animal groups and treatments timelines are shown in Fig. S2 of Supplementary material. Daily EGCG treatments were provided orally in the drinking water at a dose of 40 mg/kg (free or encapsulated). All animals were kept under controlled temperature, humidity and light conditions and given access to food and water ad libitum. Every effort was made to reduce the number of animals and minimize animal suffering. Mice were treated in accordance with the European Community Council Directive 86/609/EEC, the procedures established by the Department d'Agricultura, Ramaderia i Pesca of the Generalitat de Catalunya and approved by the local ethical committee (University of Barcelona).

### 2.13 In vivo biodistribution of EGCG/AA NPs

WT mice were given a single dose of 40 mg/kg of EGCG/AA NPs-Rho (expressed in EGCG concentration) or a corresponding dose of free Rhodamine (2.7 mg/kg) by oral gavage. After 24 h they were divided in two groups and sacrificed by: (i) cardiac perfusion with PFA 4% (previously anesthetized by intraperitoneal (i.p.) injection of sodium pentobarbital 80 mg/kg) and (ii) cervical dislocation. From the first group, brain coronal sections of 20 μm of thickness were cut on a cryostat (Leica Microsystems, Wetzlar, Germany) for rhodamine visualization under an epifluorescence microscope (Olympus BX61, Barcelona, Spain). From the second group, different organs were collected and processed to determine the biodistribution of EGCG/AA NPs by detecting the EGCG amount by LC-MS-MS as described elsewhere [46]. All measurements were run in triplicate.

### 2.14 Pharmacokinetic assay

40 mg/kg of EGCG/AA NPs (expressed as EGCG concentration) and free EGCG were administered to WT mice by oral gavage. To evaluate the pharmacokinetic profile, animals were anesthetized with isoflurane and sacrificed by cardiac puncture at different time intervals. Blood and brain were collected to measure EGCG amount by LC-MS-MS. Samples processing was performed as described Chen et al. with some modifications [47]. Briefly, blood samples were centrifuged at 4.000 *g* during 4 min at 4 °C, plasma was removed, extracted with ethyl acetate twice and resuspended in 20% AA, 10% acetonitrile. Brains were weighed, homogenized in 20% AA, 0.5 mg/ml Na_2_EDTA, PBS 0.4 M and centrifuged at 16.000 *g* during 10 min at 4 °C. Then, supernatant was extracted the same as those plasma samples. All measurements were run in triplicate.

### 2.15 Behavioural test

#### 2.15.1 Morris water maze

Morris water maze (MWM) test was performed in a circular tank with latex white water and a platform, which was kept in the same position during all the experiment, submerged 1 cm below the water surface. Temperature and intensity of light were kept at 25 ± 2 °C and 30 lx throughout all the days. The procedure was based on six training days (1 min per trial) and one test day. On the test day, the platform was removed and the mouse was placed in the tank in the quadrant opposite to where the platform used to be for 1 min. The behavioural data were acquired and analysed using SMART V3.0 (Panlab Harvard Apparatus, Germany) video tracking system.

#### 2.15.2 Novel object recognition

Novel object recognition (NOR) test was used as indicator of cognitive impairment [48]. Briefly, in a first phase of habituation (days 1–3), mice were placed in an open field (without any object) to familiarize themselves with. On day 4, two identical objects were placed at equidistance and the exploration time of each one was recorded. On the day of the test the same process was repeated by changing one of the familiar objects to a different one (novel object). The duration of each trial was 10 min. The results of the test were expressed as (eq. 2):

(2) $$\text{Exploration time}\;\left(\%\right)=\frac{\text{Exploration time of}\;\mathrm{new}\;\text{object}\;\left(s\right)}{\text{Total exploration time}\;\left(s\right)}\cdot 100$$

#### 2.15.3 Immunohistochemistry and Thioflavin-S staining

At the end of treatments and behavioural studies, mice were sacrificed by i.p. injection of ketamine/xylazine (100/10 mg/kg, respectively) and perfusion with 4% PFA. Brains were removed and maintained at 4 °C in 30% sucrose, 4% PFA solution until they were cut in 20 μm coronal sections using a cryostat (Leica Microsystems, Wetzlar, Germany). Sections were stained as described elsewhere [40]. Primary polyclonal antibodies against glial fibrillary acidic protein (GFAP) (1:1000; Dako Chemicals, Glostrup, Denmark) and Synaptophysin (SYN) (1200, Dako Products, Sta. Clara, CA, USA) and secondary antibodies AlexaFluor 594 goat anti-rabbit (Red, 1:1000; Life Technologies, Cambridge, UK) and AlexaFluor 488 goat anti-mouse (Green, 1:1000; Life Technologies, Cambridge, UK), were used. Image acquisition was carried out with an epifluorescence microscope (BX41, Olympus, Germany).

To detect Aβ plaques in brain slides, Thioflavin-S (ThS) staining was performed as described elsewhere [49]. Briefly, brain sections were incubated with 5 ml of ThS 0.3% and washed twice with 10 ml of EtOH 50% and PBS. Slides were mounted with Fluoromount on gelatin-coated glass microscope and observed under the epifluorescence microscope. Plaque quantification was carried out using the same areas, focusing on the hippocampus and cortical area.

#### 2.15.4 Evaluation of β-amyloid peptides levels

Measurement of Aβ_(1-42)_ peptides in cortical tissues was carried out using a commercially available human ELISA according to manufacturer's guideline kit (Cat # KHB3441; Invitrogen, Camarillo, CA, USA). Aβ content of cortical extracts homogenates was expressed in picograms of Aβ per milligrams of total protein [45].

#### 2.15.5 Statistical analysis

Data are presented as the mean ± S.D. One-way ANOVA followed by Tukey post hoc test was performed for groups comparison using GraphPad 6.0 Prism. Statistical significance was set at p˂0.05 (*).

## 3 Results

### 3.1 Optimization and physicochemical characteristics of EGCG/AA NPs

**Fig. 1:**
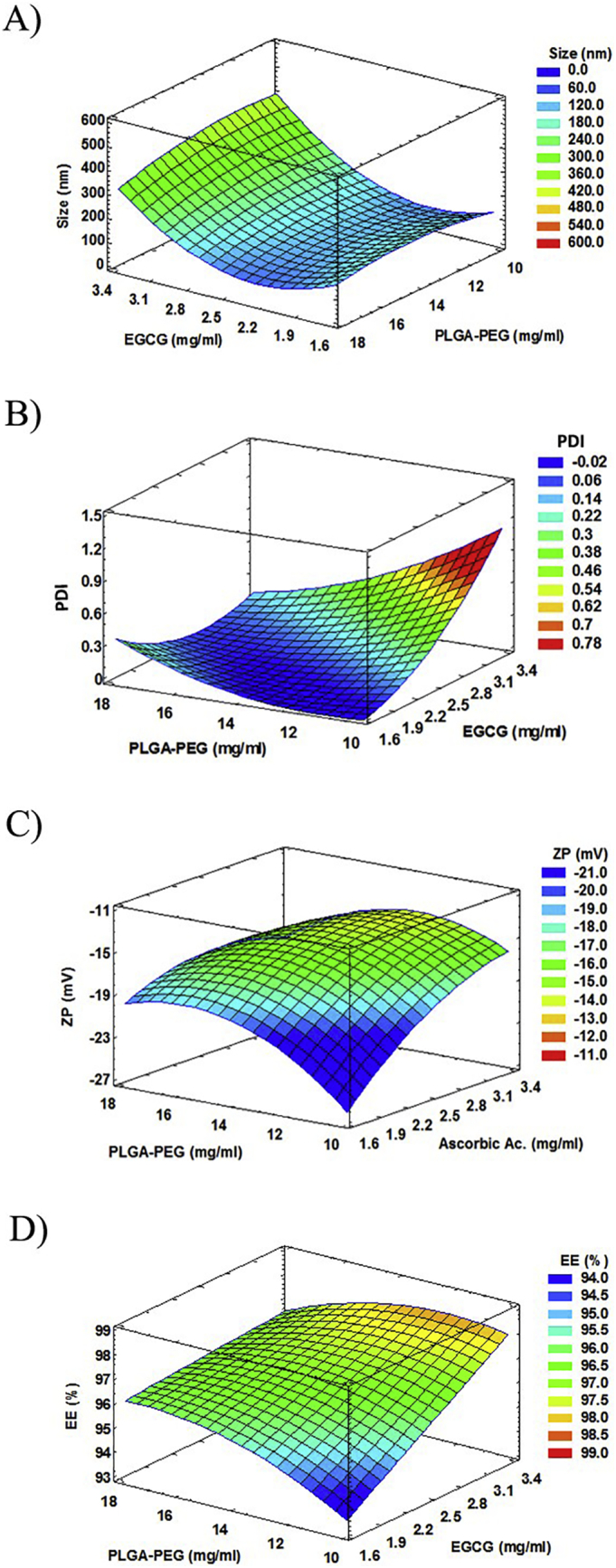
Results of EGCG/AA NPs optimization study. (A) Response surface of size at fixed value of AA 2.5 mg/ml. Optimized value 124.8 ± 5.2 nm. (B) Response surface of PDI at fixed value of AA 2.5 mg/ml. Optimized value 0.054 ± 0.013. (C) Response surface of ZP at fixed value of EGCG 2.5 mg/ml. Optimized value −15.1 ± 1.7 mV. (D) Response surface of EGCG EE at fixed value of AA 2.5 mg/ml. Optimized value 97.1 ± 2.4 (%).

Physicochemical characteristics of 16 formulations tested in the 2^3^ composite central factorial design are shown in Table 1. From this an optimal formulation was chosen consisting of 2.5 mg/ml of EGCG, 2.5 mg/ml of AA and 14 mg/ml of PLGA-5% PEG. Tween®80 concentration of the aqueous phase W_2_ and outer covering was 2.5 and 0.04% (i.e. resulting in a final concentration of 1.016% of surfactant). EGCG/AA NPs optimized in this way exhibited a monodiperse population (PDI 0.054 ± 0.013) with a Z_av_ of 124.8 ± 5.2 nm. The response surface showed a direct relationship of EGCG concentration and Z_av_ and PDI; both decreasing as the amount of EGCG decreased (Fig. 1A and B). The optimized formulation showed a ZP of ca. −15 mV (Fig. 1C). EGCG EE was higher than 97%, slightly decreasing at low concentrations of PLGA-PEG (Fig. 1D). AA EE value was higher than 94%.

**Table 1:** Optimization study results of EGCG/AA NPs by 2^3^ central composite factorial design.

Factorial points	C.EGCG		C. AA		C.PLGA-PEG		Z_av_ (nm)	PDI	ZP (mV)	EGCG EE (%)	AA EE (%)
	Coded level	(mg/ml)	Coded level	(mg/ml)	Coded level	(mg/ml)					
F1	−1	2	−1	2	−1	12	126.5 ± 4.1	0.063 ± 0.019	−17.8 ± 0 0.5	94.4 ± 4.5	91.1 ± 2.1
F2	1	3	−1	2	−1	12	174.7 ± 11.4	0.060 ± 0.004	−19.9 ± 0.6	97.5 ± 3.2	90.5 ± 6.1
F3	−1	2	1	3	−1	12	121.7 ± 0.9	0.070 ± 0.001	−13.6 ± 0.8	96.1 ± 1.2	90.3 ± 2.7
F4	1	3	1	3	−1	12	185.1 ± 8.2	0.586 ± 0.085	−16.1 ± 2.1	97.8 ± 1.3	92.5 ± 1.1
F5	−1	2	−1	2	1	16	126.5 ± 6.1	0.067 ± 0.003	−14.5 ± 0.8	96.5 ± 1.3	94.2 ± 4.2
F6	1	3	−1	2	1	16	144.6 ± 27.1	0.255 ± 0.110	−20.7 ± 2.3	97.9 ± 2.8	95.1 ± 1.2
F7	−1	2	1	3	1	16	134.1 ± 5.0	0.050 ± 0.012	−14.9 ± 1.6	96.8 ± 2.4	95.8 ± 3.1
F8	1	3	1	3	1	16	176.5 ± 18.9	0.183 ± 0.098	−17.1 ± 3.5	98.0 ± 0.8	97.0 ± 1.3
Axial points											
F9	−1.68	1.66	0	2.5	0	14	119.6 ± 5.9	0.076 ± 0.006	−12.6 ± 0.8	95.9 ± 4.5	93.9 ± 1.2
F10	1.68	3.34	0	2.5	0	14	512.4 ± 64.0	0.293 ± 0.126	−11.5 ± 0.8	97.2 ± 2.6	95.5 ± 2.1
F11	0	2.5	−1.68	1.66	0	14	277.4 ± 20.9	0.284 ± 0.207	−18.2 ± 0.8	96.4 ± 1.9	90.6 ± 2.4
F12	0	2.5	1.68	3.34	0	14	135.0 ± 10.1	0.062 ± 0.015	−12.5 ± 0.1	97.1 ± 1.3	95.3 ± 0.6
F13	0	2.5	0	2.5	−1.68	10.64	186.7 ± 2.3	0.169 ± 0.064	−14.1 ± 1.6	96.3 ± 2.1	90.1 ± 2.4
F14	0	2.5	0	2.5	1.68	17.36	129.7 ± 4.3	0.061 ± 0.006	−18.3 ± 0.4	95.7 ± 3.1	96.9 ± 0.7
Central points											
F15	0	2.5	0	2.5	0	14	124.8 ± 5.2	0.054 ± 0.013	−15.1 ± 1.7	97.1 ± 2.4	94.3 ± 1.6
F16	0	2.5	0	2.5	0	14	129.6 ± 5.2	0.063 ± 0.013	−15.7 ± 1.7	96.5 ± 2.4	96.1 ± 1.7

### 3.2 Interaction studies

No covalent bonds were observed between both drugs and polymer in line with our previous studies [40]. EGCG FTIR profile exhibited typical peaks at 1543, 1690 and 3356 cm^−1^ which correspond to C—C stretch in aromatic ring, a C=O group that links the trihydroxybenzoate and chroman groups and a OH group attached to the aromatic ring, respectively [50]. AA exhibited intense bands at 1322 and 1674 cm^−1^, which correspond to the enol hydroxyl group and the stretching vibration of the C=C group, respectively [51]. PLGA-PEG showed a peak at 1743 cm^−1^ corresponding to the C—O stretching vibration of the carbonyl groups of lactic and glycolic acid. Intense bands were also detected between 2900 and 2950 cm^−1^ due to the C—H stretching of PLGA-PEG [52]. Drug-loaded NPs exhibited a very similar FTIR profile to polymer, as well as empty NPs, but not as EGCG FTIR pattern. The encapsulation of the drug was evident by an important decrease of the absorbance in the EGCG/AA NPs profile against the polymer profile (Fig. S3A of Supplementary material).

EGCG and AA exhibited similar DSC profiles due to their common crystalline chemical structure (Fig. S3B of Supplementary material). Endothermic peaks of melting transition at 225 and 195 °C, respectively, more intense in the case of AA, were observed. These peaks were followed by an exothermic peak at 235 °C in both cases, which is related to the melting process of the drugs. Drug-loaded NPs and empty NPs DSC profiles were similar to that of polymer itself, suggesting that both molecules may converge to an amorphous or disordered crystalline form which led to their inclusion inside the NPs [50].

These results suggest that both EGCG and AA were dissolved in the form of a molecular dispersion and successfully included into the PLGA-PEG matrix.

### 3.3 NP formulated EGCG displays improved physicochemical stability

**Fig. 2:**
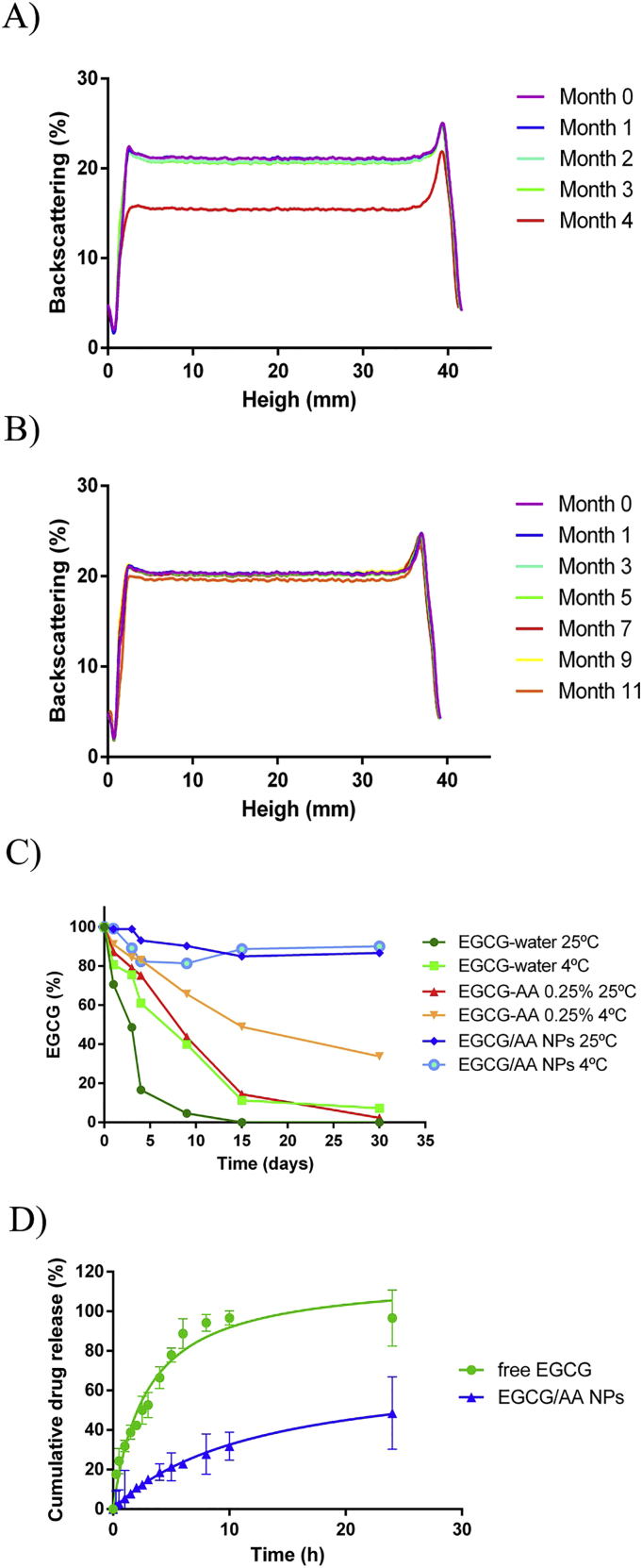
Stability and in vitro drug release results. (A) Backscattering profile of EGCG/AA NPs at 25 °C showed nanovehicle instability after 4 months. (B) Backscattering profile of EGCG/AA NPs stored at 4 °C did not show any instability for up to 11 months. (C) EGCG chemical stability in different solutions, formulations and storage temperatures. EGCG showed a significant increase in the stability after the addition of AA to the medium and its incorporation into PEGylated PLGA NPs. (D) In vitro drug release of EGCG/AA NPs and free EGCG. In each condition 25 mg of EGCG were dialysed in a total volume of 150 ml. Thus 0.167 mg/ml was 100% of the maximal equilibrium concentration. Cumulative drug release from EGCG/AA NPs at 24 h = 48.6%. Data shown was from triplicate experiments.

EGCG/AA NPs were stored at either 4 °C and 25 °C for extended periods. Backscattering profiles were established to determine stability (Fig. 2A and B). The formulation only became instable after 4 mo of storage at 25 °C. At this time, it was also noted that coloration of the normally white sample turned brown, presumably due to the oxidation of both drugs. Furthermore a precipitate appeared presumably due to degradation of the polymeric matrix [53] (Fig. S4 of Supplementary material). By contrast, ECGC/AA NPs stored at 4 °C remained stable for up to 11 months. Z_av_, PDI and ZP measurements confirmed these results (Table S1 of Supplementary material).

HPLC measurements showed that free EGCG suffered fast degradation when it was dissolved in aqueous solutions. The half-life of EGCG in water was ca. 3 and 8 days at 25 °C and 4 °C, respectively, in accordance with a previous report by Proniuk et al. [54]. The addition of AA 0.25% increased half-life of the drug to ca. 9 and 15 days at 25 °C and 4 °C, respectively. In contrast, EGCG incorporated in EGCG/AA NPs maintained at least 80% of its integrity for 1 mo at both temperatures (Fig. 2C).

### 3.4 In vitro drug release profile

One of the main advantages of polymeric controlled drug delivery systems is sustained release of encapsulated drugs, which is usually governed by a diffusion/degradation process [53]. The drug release profile of EGCG/AA NPs was determined in vitro by dialysis against a release medium at acid pH, taking into consideration the EGCG instability at neutral pH and, in preparation of oral use of EGCG/AA in mice (see below), the acid environment of the stomach [[55], [56], [57]]. When free EGCG was dialysed, its concentration in the dialysate reached the theoretical equilibrium concentration after about 10 h (Fig. 2D). In contrast only 23% of EGCG had been released from EGCG/AA NPs to the dialysate at that time. After 24 h, NPs had released almost 50% of EGCG. Taken together this indicated that our NP formulation displayed characteristics of sustained release.

### 3.5 In vivo biodistribution and EGCG pharmacokinetics

**Fig. 3:**
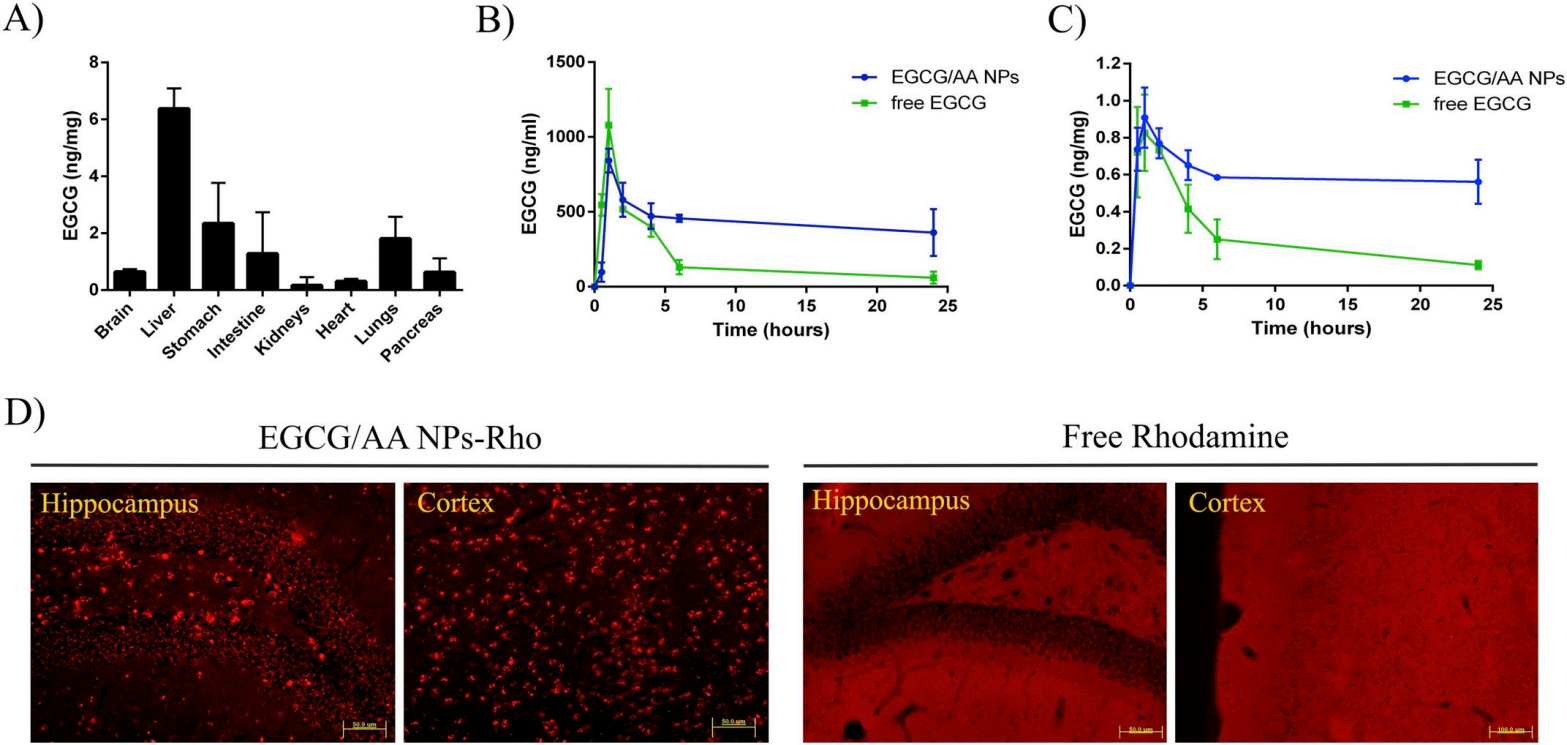
In vivo biodistribution and pharmacokinetics of EGCG/AA NPs. 3 months-old C57BL/6 WT mice were treated with a single oral dose of EGCG/AA NPs and EGCG/AA NPs-Rho 40 mg/kg. (A) Histograms show biodistribution of EGCG in brain, liver, stomach, intestine, kidneys, heart, lungs and pancreas of mice after 24 h of EGCG/AA NPs administration. Data expressed as EGCG amount per mg of tissue. Mean administered volume of EGCG/AA NPs was 448 μl (1.120 mg). Mean brain EGCG concentration was 0.6 ng/ml. With mean brain weight of 470 mg this represented 0.025% of administered EGCG. (B) Plasma and (C) brain pharmacokinetic profile of EGCG following single oral dose administration of free EGCG or EGCG/AA NPs (40 mg/kg). Data expressed as EGCG amount per ml of plasma and EGCG amount per mg of tissue, respectively. (D) Rhodamine detection in the *dentatus gyrus* of the hippocampus and the cortex of mice treated with EGCG/AA NPs-Rho (40 mg/kg) or free Rhodamine (2.7 mg/kg). Scale bar 50 μm.

3 months-old C57BL/6 mice were treated with and single oral dose of EGCG/AA NPs 40 mg/kg. After 24 h EGCG content was determined in various organs (Fig. 3A). EGCG had accumulated strongly in the liver. Whilst in the brain, EGCG concentration was ca. 10 times lower, brain accumulation constituted ca. 0.025% of the administered EGCG. Pharmacokinetic profiles of EGCG were established after an oral administration both of EGCG/AA NPs and free EGCG (Fig. 3B and C, Table 2). After 1 h, maximal EGCG plasma concentrations were very similar under both conditions (1078.976 ± 242.238 ng/ml for free EGCG v. 842.590 ± 78.911 ng/ml for EGCG/AA NPs). Thereafter, whilst a rapid decrease in plasma EGCG concentration was observed in mice administered with free EGCG (to 60.267 ± 40.150 ng/ml after 24 h), EGCG plasma concentration stabilised at ca. 500 ng/ml (with 361.346 ± 156.176 ng/ml at 24 h) in mice treated with EGCG/AA NPs. Analysis of AUC of plasma EGCG concentrations revealed significant differences with 10,270.624 ± 123.564 h.ng/ml following oral treatment with EGCG/AA NPs and only 4509.790 ± 65.411 h.ng/ml following free EGCG.

**Table 2:** Pharmacokinetic parameters of an oral gavage administration of EGCG/AA NPs and free EGCG 40 mg/kg in 3 months-old C57BL/6 mice. Non-compartmental model.

Parameter	EGCG/AA NPs	Free EGCG	Unit	EGCG/AA NPs	Free EGCG	Unit
	Plasm			Brain		
AUC^a^	10270.624± 123.564	4509.790 ± 65.411	h.ng/ml	14.410 ± 3.511	6.426 ± 0.811	h.ng/mg
C_max_	842.590 ± 78.911	1078.976 ± 242.238	ng/ml	0.908 ± 0.162	0.826 ± 0.206	ng/mg
T_max_	1.000 ± 0.000	1.000 ± 0.000	h	1.000 ± 0.000	1.000 ± 0.000	h
ʎ_z	0.013 ± 0.007	0.0871 ± 0.006	1/h	0.005 ± 0.003	0.058 ± 0.006	1/h
HL ʎ_z	52.360 ± 9.024	7.956 ± 0.625	h	128.144 ± 16.051	12.025 ± 1.009	h
Vz/F	1850.574 ± 75.166	2503.858 ± 100.021	ml	NA	NA	ml
Cl/F	24.498 ± 7.212	218.1483 ± 46.854	ml/h	NA	NA	ml/h
MRT∞	75.415 ± 6.341	9.489 ± 0.079	h	184.687 ± 11.267	14.860 ± 2.362	h

a AUC was measured between 0 and 24 h.

A similar pharmacokinetic profile was observed in the brain of these mice. EGCG reached maximal concentration of 0.908 ± 0.162 ng/mg when it was administered encapsulated in NPs, as compared to 0.826 ± 0.206 ng/mg following free form administration. Again cerebral EGCG concentrations stabilised at a significantly higher concentrations following EGCG/AA NPs, with EGCG concentrations of 0.561 ± 0.119 ng/mg and 0.111 ± 0.022 ng/mg at 24 h, following treatment with EGCG/AA NPs and free EGCG, respectively. AUC analyses showed 14.410 ± 3.511 and 6.426 ± 0.811 h.ng/mg following encapsulated and free administration, respectively. Overall these data indicated that NPs stabilised circulating EGCG and consequently cerebral accumulation of the drug.

EGCG brain penetration was investigated indirectly by histochemical analysis in mice following a single oral dose of EGCG/AA NPs-Rho. Inspection of fixed coronal sections revealed discrete accumulation of Rhodamine structures, which were ca. 5–15 μm in size in various areas of the brain (Fig. 3D) (shown are the *dentatus gyrus* of hippocampus and cortex). In contrast, mice treated with free Rhodamine showed a diffused staining in the brain.

### 3.6 Effect of EGCG/AA NPs on BBB integrity

**Fig. 4:**
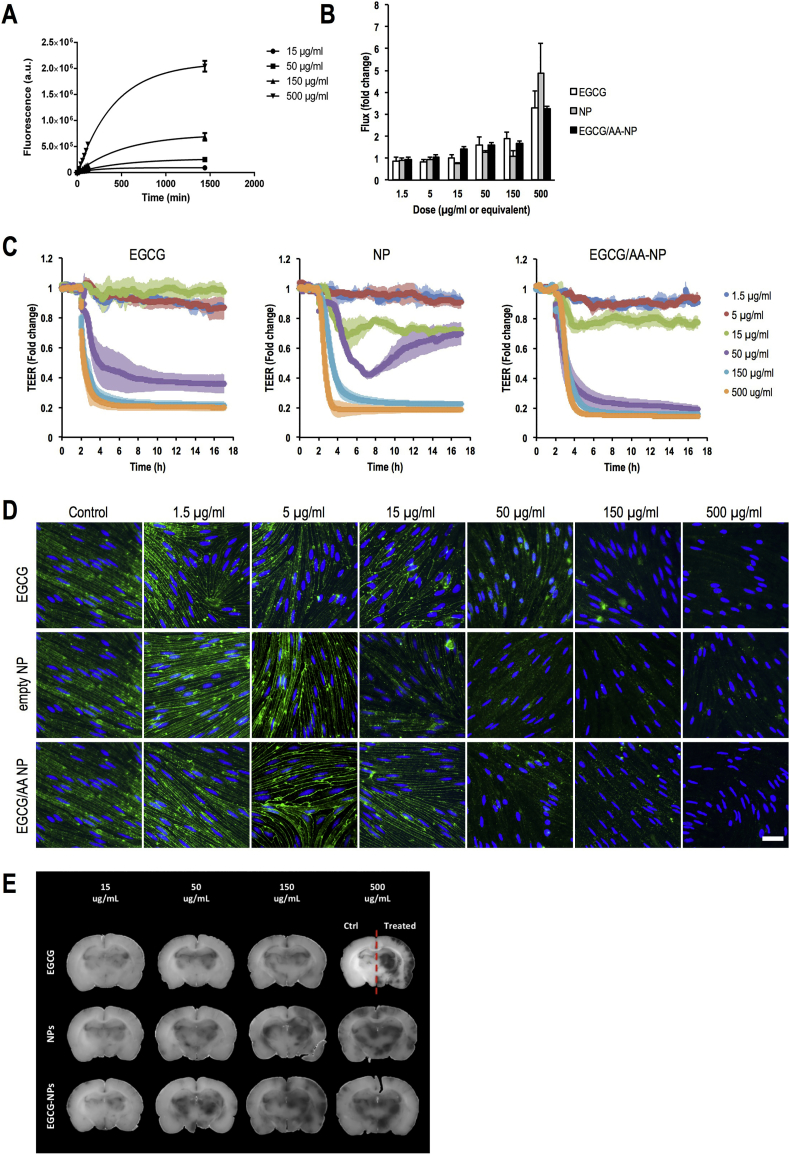
Effect of EGCG formulations on in vitro models (A) Flux of 15, 50, 150 and 500 μg/ml of EGCG/AA NPs-Rho across primary rat BMVECs within the initial 2 h. Data are showed as mean ± SD. At 1440 min values of each data set showed statistical difference to each of the other groups (p < 0.001, ANOVA). (B) FITC-dextran flux across primary rat BMVECs in the presence of 1.5, 5, 15, 50, 150 and 500 μg/ml EGCG, EGCG/AA NPs and equivalent amounts of empty NPs. Flux rates in the absence of drug were normalised to 1. Data are showed as mean ± SEM. (C) Normalised TEER real time measurement of BMVCEs monolayer in response to 1.5, 5, 15, 50, 150 and 500 μg/ml EGCG, EGCG/AA NPs and equivalent amounts of empty NPs (added at 2 h). Shown are mean ± SEM. (D) Claudin-5 (green) and DNA (blue) staining of BMVECs monolayer after exposure to 1.5, 5, 15, 50, 150 and 500 μg/ml EGCG, EGCG/AA NPs and equivalent amounts of empty NPs for 1 h. Scale bar 10 μm. (E) Evans Blue/Albumin leakage response in ex vivo rat brains treated on the indicated side with 1.5, 5, 15, 50, 150 and 500 μg/ml EGCG, EGCG/AA NPs or equivalent amounts of empty NPs for 1 h. (For interpretation of the references to color in this figure legend, the reader is referred to the web version of this article.)

NPs have been proposed to facilitate or even open a specific transport route across the BBB [58,59]. Passage of EGCG/AA NPs-Rho was assessed using rat primary BMVECs, a well-established in vitro model of BBB [41]. Added apically at concentrations of 15 of 15, 50, 150 and 500 μg/ml EGCG/AA NPs-Rho crossed BBB cell monolayers in concentration dependent manner (Fig. 4A), initially being mostly linear but then plateauing when reaching the equilibrium concentration (kinetically fitting an exponential one-phase association model).

Free EGCG at and above 15 μg/ml, EGCG/AA NPs at and above EGCG concentration of 15 μg/ml as well as empty NPs at equivalent concentrations induced significantly enhanced 4 kDa dextran flux in cultured primary rat BMVECs (Fig. 4B). They also induced disruption of BMVEC electrical barrier indicating that tight junctions were affected (Fig. 4C). Indeed, immunohistochemical analysis showed that junctional Claudin-5 was lost from primary BMVEC monolayers treated with increasing concentrations of free EGCG, empty and EGCG/AA NPs (Fig. 4D). Excessive leakage was also observed in rat ex vivo brains in response to free EGCG, empty NPs and EGCG/AA NPs at and above concentrations (or equivalents) of 150 μg/ml (Fig. 4E), indicating that EGCG and empty NPs themselves could disrupt the BBB.

### 3.7 EGCG/AA NPs increase synaptic expression in APP/PS1 mice

**Fig. 5:**
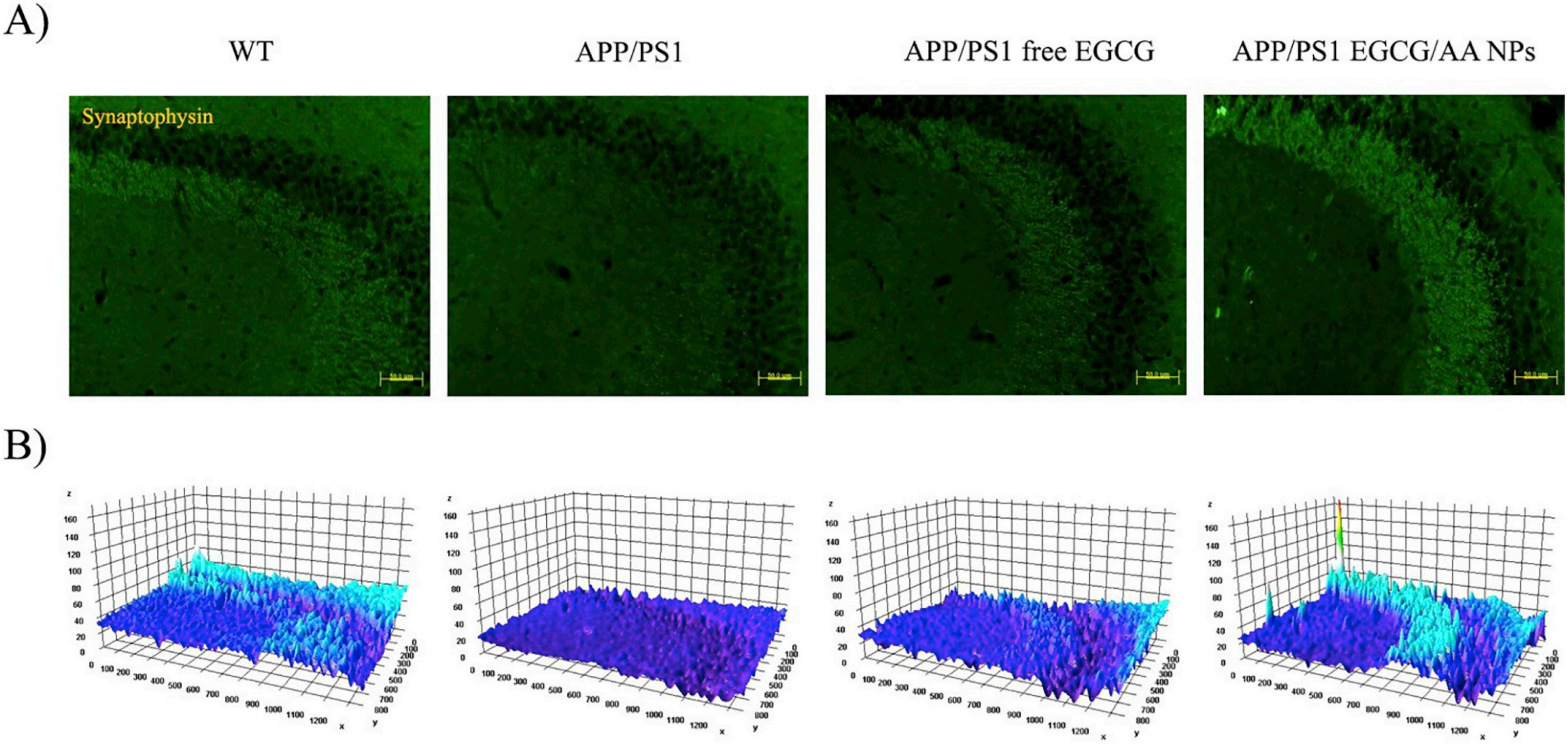
Effect of EGCG treatments on SYN expression. 3 months-old APP/PS1 mice were orally treated with EGCG/AA NPs or free EGCG 40 mg/kg/day for 3 months. At 6 months, animals were sacrificed by perfusion with 4% PFA and brains were cut with a cryostat. SYN immunostaining was performed on 20 μm coronal sections. (A) SYN staining of CA3 hippocampus region. Scale bar 50 μm. (B) 3D surface mapping analysis of SYN staining. Interactive 3D surface Plot v 2.4, Image J. Zones with synaptic labeling of the EGCG/AA NPs treated mice exhibited a tridimensional relief and color intensity higher to those of the WT group.

We next investigated the therapeutic effect of EGCG/AA NPs in APP/PS1 mice. Synaptic loss, as evidenced by decreased SYN immunoreactivity in the hippocampus area of patients with early and stablished AD and demented individuals, correlates with the development of neuropathology and severe cognitive deficit [60]. APP/PS1 mice showed reduction of SYN staining in the CA3 region of hippocampus. In contrast, APP/PS1 mice, treated with either free or NPs formulated EGCG, displayed enhanced SYN staining. In fact, EGCG/AA NPs group showed even higher SYN staining than the WT group (Fig. 5A). 3D surface mapping analysis confirmed these results (Fig. 5B).

### 3.8 EGCG/AA NPs reduce neuroinflammation and Aβ plaque/peptide burden in APP/PS1 mice

**Fig. 6:**
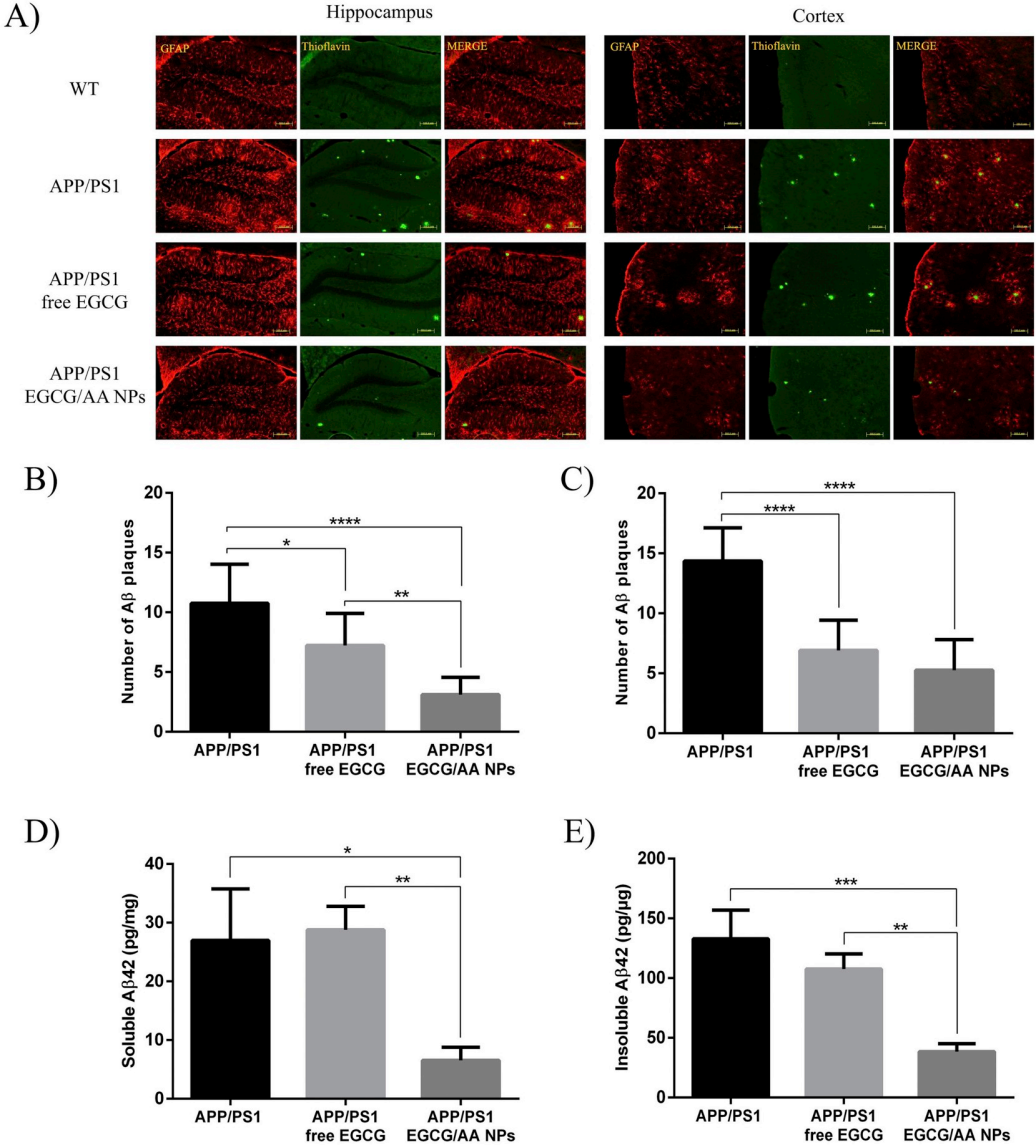
Effect of EGCG treatments on neuroinflammation and Aβ plaque/peptide burden. (A) 3 months-old APP/PS1 mice were orally treated with EGCG/AA NPs or free EGCG 40 mg/kg/day for 3 months. At 6 months, animals were sacrificed by perfusion with 4% PFA and brains were cut with a cryostat. GFAP and ThS immunostaining was performed on 20 μm coronal sections. *Dentate gyrus* of hippocampus and cortex area are shown. Scale bar 100 μm. Images of ThS staining of 100 μm coronal sections of perfused animals were used for Aβ plaques depositions count. Histograms show Aβ plaques depositions count at (B) hippocampus and (C) cortex area. In ELISA kit, animals were sacrificed by cervical dislocation, and brain cortices were homogenized. Histograms show (D) soluble and (E) insoluble amounts of Aβ_(1–42)_ peptide expressed as pg/mg of total protein. ANOVA analysis of data is included in the table S2 of the supplementary material.

Neuroinflammation with accompanying accumulation of Aβ plaques plays a crucial role in the pathogenesis of AD [61]. Astrocyte activation and concomitant enhanced GFAP expression have been widely used as a neuroinflammatory indicator [62]. Neuroinflammation and Aβ plaque accumulation was assessed in WT and APP/PS1 mice by Th—S and GFAP staining (Fig. 6A). Hippocampal and cortical areas of APP/PS1 mice displayed much stronger GFAP reactivity as well as altered morphology when compared to that of WT littermates. Treatment of APP/PS1 mice with free EGCG had little effect on GFAP expression. However, EGCG/AA NPs treatment strongly reduced GFAP reactivity, with observed levels similar to those of WT littermates. APP/PS1 mice showed Aβ plaques accumulation in these cerebral areas analysed. Whilst treatment with either free or NP encapsulated EGCG showed reduction of Aβ plaques in the hippocampus, the reduction was significantly greater in the EGCG/AA NPs treated group (Fig. 6B). Cortical Aβ plaque count revealed that both treatments were equally effective in reducing the number of Aβ plaques (Fig. 6C).

Soluble and insoluble Aβ42 peptide was quantified in cortical extracts of APP/PS1 mice by ELISA. Oral EGCG/AA NPs treatment led to a significant reduction of both soluble and insoluble Aβ42 peptide in the cortex (Fig. 6D and E). However, treatment with free EGCG did not show any significant reduction in either Aβ42 peptide population. Taken together these data indicated that EGCG/AA NPs were more effective in reducing neuroinflammation and pathological Aβ accumulation in APP/PS1 mice.

### 3.9 EGCG/AA NPs improve EGCG effectiveness on spatial memory and learning process in APP/PS1 mice

**Fig. 7:**
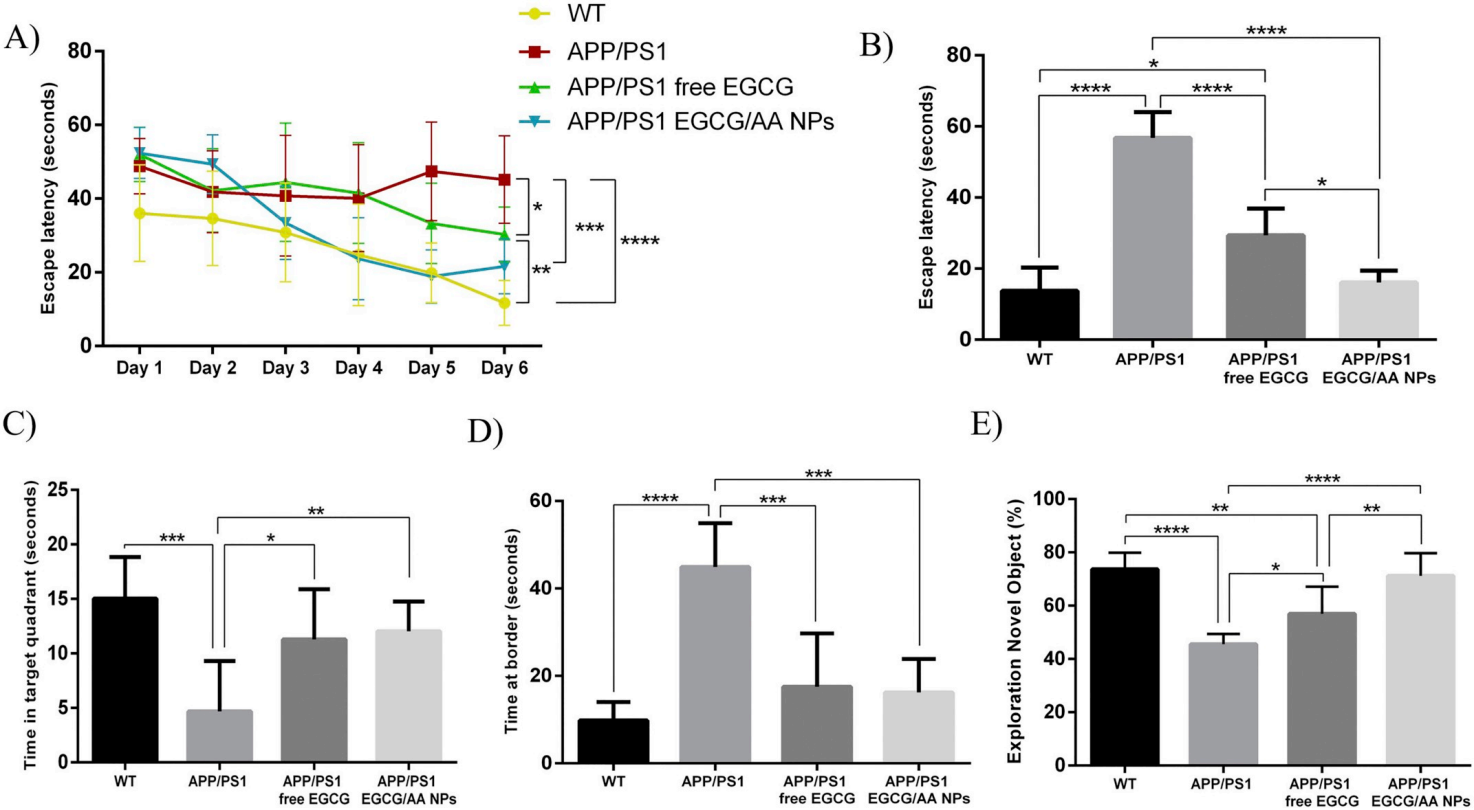
Behavioural tests results. 3 months-old APP/PS1 mice were orally treated with EGCG/AA NPs or free EGCG 40 mg/kg/day for 3 months and then subjected to MWM and NOR tests. (A, B) Histograms shows the escape latency of learning process (statistics at the end of the training phase) (A) and of the test day (B) of WT, non-treated APP/PS1 and treated APP/PS1 mice. (C, D) Histograms show the time expended in the target quadrant (C) and the time expended at border area (D) the test day. (E) Histograms show the time percentage of investigation of the novel object by WT, non-treated APP/PS1 and treated APP/PS1 mice in the NOR test. ANOVA analysis of data is included in the table S3 of the Supplementary material.

The behaviour of APP/PS1 transgenic mice was assessed following EGCG treatment for 3 mo. Mice were subjected to a MWM test as measure for learning ability and spatial memory mechanisms [63]. APP/PS1 mice displayed significantly longer escape latency time than WT control mice. Following treatment with free or NPs formulated EGCG, APP/PS1 mice showed a significant decrease in escape latency time during the training phase (Fig. 7A). On the day of the test, EGCG/AA NPs treated APP/PS1 mice showed a significantly reduced escape latency time, which was similar to WT mice (Fig. 7B). Free EGCG treatment also significantly reduced escape latency on the test day, however it was still significantly longer than that of EGCG/AA NPs treated APP/PS1 mice. APP/PS1 also showed significantly reduced exploration time in the target quadrant. Exploration time was significantly enhanced with either EGCG treatment (Fig. 7C). Additionally, the time spent in the border area was analysed as indicator of the anxiety level. Results revealed that both treated groups significantly displayed reduced border exploration time, nearly at levels of WT controls (Fig. 7D).

### 3.10 EGCG/AA NPs improve EGCG effect on cognitive process in APP/PS1 mice

The NOR test, which is based on the natural tendency of mice to explore new objects and environments is widely used in the evaluation of short- and long-term cognitive deficits in AD [48]. APP/PS1 mice were treated with EGCG/AA NPs or free EGCG for 12 weeks. Assessment by NOR test showed a significant improvement in performance in both treated groups. Significantly, the EGCG/AA NPs group performed even better results than the free drug group, with similar NOR scores than those observed in the WT group (Fig. 7E). Taken together these data indicated that EGCG/AA NPs offered a significant treatment advantage over free EGCG.

## 4 Discussion

Neurodegeneration and dementia associated with AD is the result of many cytological and biochemical alterations in the brain [1]. There are currently no effective treatments and in this context, our study focused on developing EGCG as a new therapeutic option for AD. Specially we opted to address this drug's inherent chemical instability and resulting low bioavailability [24] by incorporating it into polymeric NPs. Nanovehicle incorporation stabilised EGCG, produced a better pharmacokinetic profile of EGCG with higher concentrations in the brain and, in APP/PS1 mice, was more effective in reducing neuroinflammation and Aβ accumulation and in improving cognitive deficits.

We chose to formulate EGCG in polymeric nanostructures of PLGA based matrix. PLGA was one of the first polymers approved by US Food and Drug Administration for biomedical applications because of its biocompatibility, biodegradability and non-toxicity [64]. Overall, the biocompatibility and safety of PLGA NPs has been demonstrated and does not involve a risk of peripheral side effects [72,73]. PLGA is widely used in nanomedicine for brain diseases, since it possesses a great versatility in its manufacture, physicochemical properties and functionalization [65]. Likewise, PEGylated polymeric matrix was used, since it is well described that the coating with PEG enhance a rapid spread of carried drugs within brain tissue [66]. Conjugation of PEG to the NPs surface may also contribute to an enhanced penetration across the intestinal barrier, since PEGylation has been described to increase the permeability coefficient of solid lipid nanoparticles by 1.5–2 times and their resulting bioavailability by 1.99 to 7.5 times [67]. Additionally, our EGCG/AA NPs were formulated with Tween®80 as surfactant (final concentration 1.016%) since this has been reported to facilitate brain uptake of a number of drugs [68]. Lastly, we chose to incorporate AA together with EGCG in NPs with the aim to provide a stabilizing, anti-oxidant environment. AA, together with EGCG, may also mitigate potential local inflammatory response [69,70], which has been ascribed to hydrolytic degradation products derived from PLGA during chronic administration [71]. We have used similar EGCG PLGA NPs before, albeit at lower drug and surfactant concentration and without AA, with demonstrable therapeutic benefits in a mouse model of epilepsy [40].

Our optimized formulation of EGCG/AA NPs significantly enhanced shelf stability of EGCG, in particular in an aqueous environment and demonstrated adequate properties for enhanced brain delivery. With our formulation, we achieved small Z_av_, monomodal population and negative surface charge, which contributes to enhance the stability of NPs due to the increase of repulsion forces. In fact, PLGA matrix commonly suffers surface erosion and is degraded through a hydrolytic cleavage of its polyester backbone, resulting in smaller structures of byproducts, which also contains encapsulated drug [74]. These processes give rise to a reduction of particle size, which could also contribute to the enhancement of encapsulated drug penetration.

Importantly, EGCG showed an adequate and sustained release profile from the polymeric nanostructures, indicating suitability for long-term treatment. Taken together EGCG/AA NPs showed the desired characteristics of enhanced stability, sustained release and physicochemical properties compatible with brain penetration.

A number of reports describe enhanced drug transport to the brain when using non-ligand coated PLGA NP [59,65,75,76]. However, the mechanism underlying these observation remains disputed [58]. Experimental data shows that PEG coating and the use of surfactants such as Tween are important for NP formulated drug passage across the BBB [66,68]. There is clear evidence that PLGA NPs with a Z_av_ of around 100 nm can be specifically transported through BBB endothelial cells by absorptive transcytosis [58,59,77]. However, it is unclear if such transcytosis events, observed at relatively scarce numbers, contribute in a quantitative manner to the measured drug concentrations in the brain [58]. In the present study we have not investigated the transport of intact NPs across the BBB directly. When using EGCG/AA NP-Rho we observed concentration-dependent passage of Rhodamine across the BBB in vitro, which appeared entirely diffusion controlled, suggesting that flux occurred through gaps in the BBB. Similarly, Rhodamine aggregates were observed in the hippocampus and the cortex when EGCG/AA NP-Rho were administered in vivo. Since Rhodamine was coupled covalently to the PLGA matrix, it is likely that intact NPs or their hydrolytic degradation products crossed the BBB. Diameters of Rhodamine clusters in the brain ranged from 5 to 15 μm, suggesting aggregation of the NPs and/or their uptake by resident or migrating phagocytic cells.

Significantly, we can report here that each of the constituent components of EGCG/AA NPs affected BBB integrity in vitro and ex vivo, suggesting that ultimately free or nanomaterial formulated EGCG penetrated the BBB through destabilized junctions, which was in complete agreement with our EGCG/AA NPs-Rho data. Whilst junction destabilization in our models was only measurable at concentrations several fold higher than what was observed in the plasma in vivo, it is likely that lower EGCG/AA NPs constituent concentrations also affected BBB integrity. In addition, given that the nanomaterial appeared aggregated when detected in the brain, it is also possible that high localized concentrations of aggregated material led to severe localized tight junction disruption and breach of the BBB similarly to what we observed in cultured BMVEC. In addition, it is well stablished that AD pathology involves a dysfunctional BBB with an enhanced permeability [78,79]. Indeed, APP/PS1 mice showed enhanced perivascular IgG accumulation indicating a dysfunctional BBB (Fig. S5 of Supplementary material). This contributes to the penetration of many molecules which would not normally cross the BBB under healthy conditions [80]. Therefore, we propose that quantitative uptake of EGCG to the brain occurred through a destabilized BBB.

Irrespective of the transport route, our study shed light on possibly the most therapeutically important property of EGCG-loaded NPs: when administered as PLGA-PEG nanomaterial, EGCG exhibited pharmacokinetic profile with significantly enhanced residence time in blood stream and brain tissue. When provided orally to mice either free or as EGCG/AA NPs, EGCG accumulated rapidly in the blood, reaching peak plasma concentrations of around 1 μg/ml within 1 h of administration. Importantly, peak concentrations measured were similar in both cases, indicating that the same absorption route from the GI tract to the blood was used and that PLGA NPs did not specifically enhance GI uptake. GI absorption of tea catechins, appears to involve active transport [81]. Importantly, longer term plasma concentrations were ca. 5 times higher in animals treated with EGCG/AA NPs, suggesting less degradation (either chemical or in the liver). This pharmacokinetic profile was mirrored in the brains of treated animals, where peak EGCG concentrations were also observed after around 1 h. Again subsequently, concentrations fell rapidly but remained much higher in animals having received EGCG/AA NPs as opposed to free EGCG. Importantly, relative brain EGCG concentrations were similar to those in the plasma (see footnote^2^), indicating that pharmacokinetic profile of EGCG in the brain was a direct consequence of that in the plasma. Overall our results strongly suggested that brain uptake of EGCG occurred through destabilized BBB endothelial junctions by diffusion and was enhanced due to greater stability and dwell time in the blood.

Previous studies have shown that different treatments with EGCG improved the memory process in AD animals models [16]. Lee et al. demonstrated this after an i.p. EGCG treatment of 3 mg/kg/day during 3 weeks in a LPS-induced memory impairment mouse model of AD [82]. We also found marked therapeutic effects of free EGCG in APP/PS1 mice using MWM and NOR behavioural test. However, overall, EGCG formulated as EGCG/AA NPs led to even better therapeutic outcome than the free drug alone. Significantly, improvements were found in spatial learning and memory (MWM test). Furthermore, NOR test also showed that EGCG/AA NPs treatment of APP/PS1 mice restored the recognition memory and enhanced cognitive process far more than free EGCG, reaching scores comparable to those of the WT group.

Improvement in brain function clearly had cytological and biochemical basis. EGCG reduced neuroinflammation and neurodegeneration in agreement with published data [16]. However, we found even greater EGCG anti-neuroinflammatory activity, as well as a significant decline in the accumulation of Aβ plaques, when EGCG was supplied as EGCG/AA NPs. These findings were in accordance with those obtained by Li et al., which showed that Aβ deposits were reduced by 60% in the frontal cortex and 52% in the hippocampus after 3 months of treatment of EGCG 20 mg/kg/day by oral gavage in an APP transgenic mice [83].

We also found a strong increase in SYN staining following treatment of APP/PS1 mice with EGCG/AA NPs, indicating protection of synapses and increase of synaptogenesis. This correlated well with the cognitive improvement exhibited in behavioural tests. Many studies have demonstrated that small increases in the levels of Aβ42 peptide lead to permanent synaptic function impairment and neuroinflammatory responses [84]. Indeed, the high level of both soluble and insoluble Aβ42 peptide in APP/PS1 mice was significantly reduced following EGCG/AA NPs, but not free drug treatment.

## 5 Conclusions

In summary, the current work demonstrates the improvement of EGCG stability and effectiveness when loaded in PEGylated PLGA NPs within an antioxidant environment. EGCG/AA NPs were able to increase drug permanence in blood stream and brain tissue, reduce the Aβ plaques burden, Aβ42 peptide levels and neuroinflammation, and enhance synaptogenesis, memory and learning process. All these facts contributed to a significant reduction of cognitive impairment in APP/PS1 mice. Based on these findings, we propose EGCG/AA NPs as a novel, safe and suitable therapeutic alternative for the treatment of AD.

## Financial support

This work was supported by the 10.13039/501100004837Spanish Ministry of Science and Innovation (MAT 2014-59134-R, SAF2017-84283-R and PI2016/01), CB06/05/0024 (CIBERNED) and the European Regional Development Founds. AC^a,b^, ME^a,b^, and MLG^a,b^ belong to 2017SGR-1477. ME^c,d,e^, CA^f^, and AC^c,d^ belong to 2014SGR-525.

## Conflict of interest

None of the authors have any conflicts of interest including any financial, personal or other relationships with other people or organizations. All authors have reviewed the contents of the manuscript being submitted, approved its contents and validated the accuracy of the data.

## Appendix A Supplementary data

Supplementary material Image 1

## Footnotes

2 Assuming a density of 1.04 g/ml, mean brain concentration was ca. 624 ng/ml, which was very similar to mean blood concentration (see Fig. 3B and C).
