# Supplementary material for: Dual-drug loaded nanoparticles of Epigallocatechin-3-gallate (EGCG)/Ascorbic acid enhance therapeutic efficacy of EGCG in a APPswe/PS1dE9 Alzheimer's disease mice model
Source: J Control Release. 2019 May 10;301:62–75. doi: 10.1016/j.jconrel.2019.03.010 (PMC6510952; doi:10.1016/j.jconrel.2019.03.010)
Supplement: Supplementary file 1 — Supplementary material [file mmc1.pdf]

Supplementary material

Dual-drug loaded nanoparticles of Epigallocatechin-3-gallate (EGCG) / Ascorbic acid enhance therapeutic efficacy of EGCG in a APPswe/PS1dE9 Alzheimer's disease mice model.

Cano *et al.*

Figures

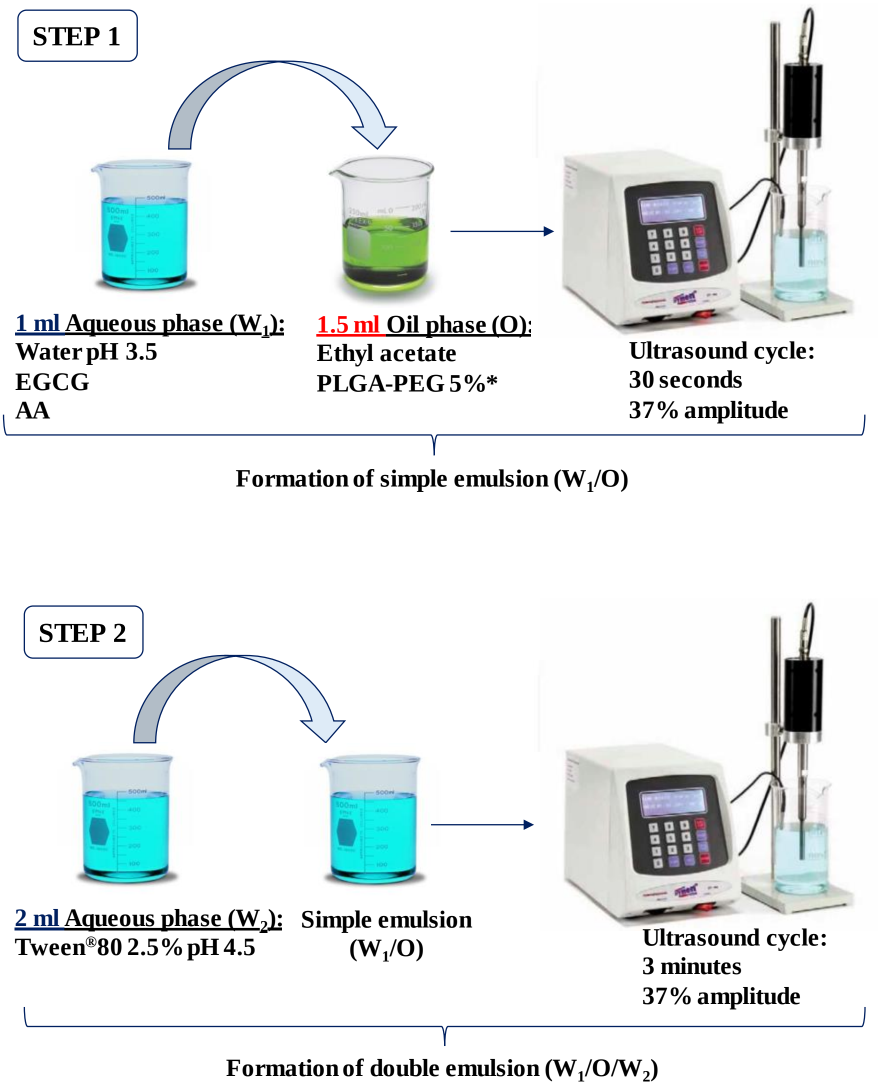

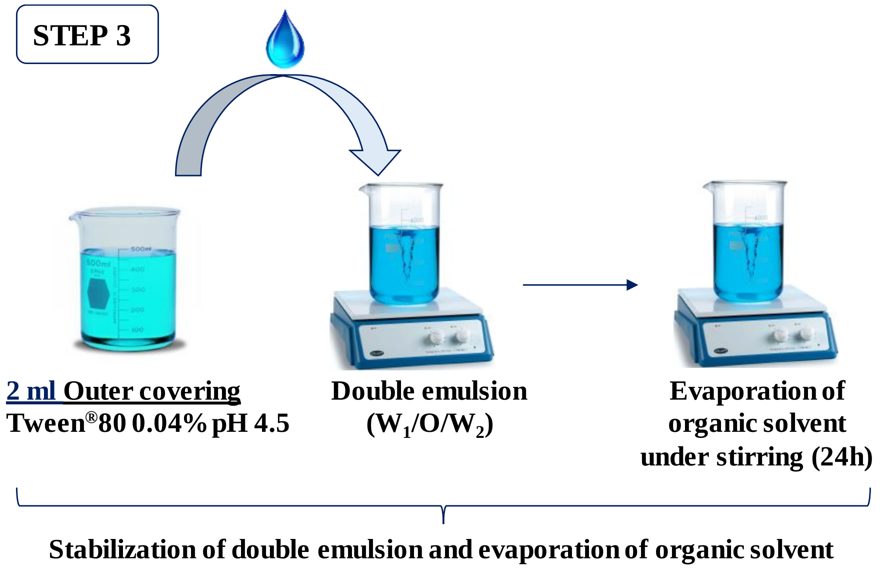

Figure S1. Fabrication of EGCG/AA NPs by double emulsion method. Final volume of 5 ml. *Percentage of PEG that contains PLGA.

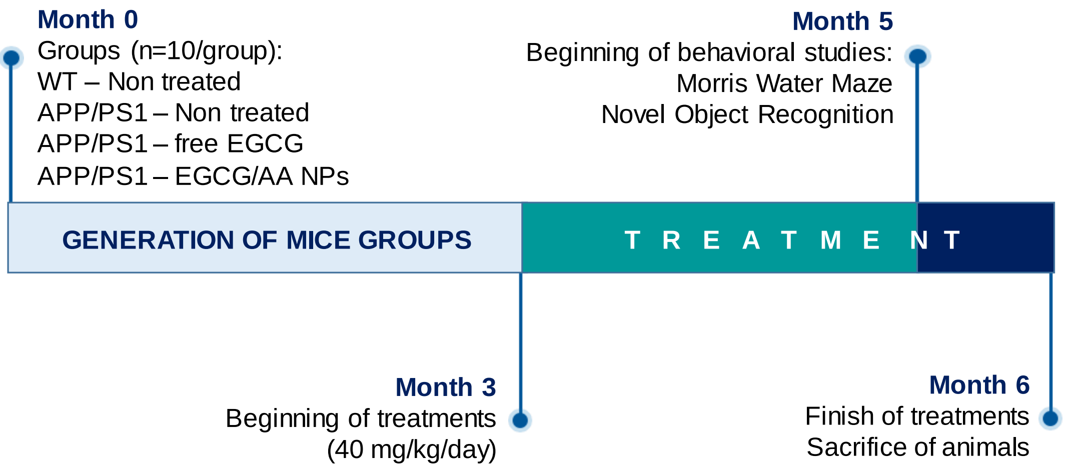

Figure S2. Timeline, groups distribution, dosage and behavioural tests of EGCG/AA NPs and free EGCG treatments. Dose of both treatments were recalculated and readjusted every week according to the weight gain of the animals.

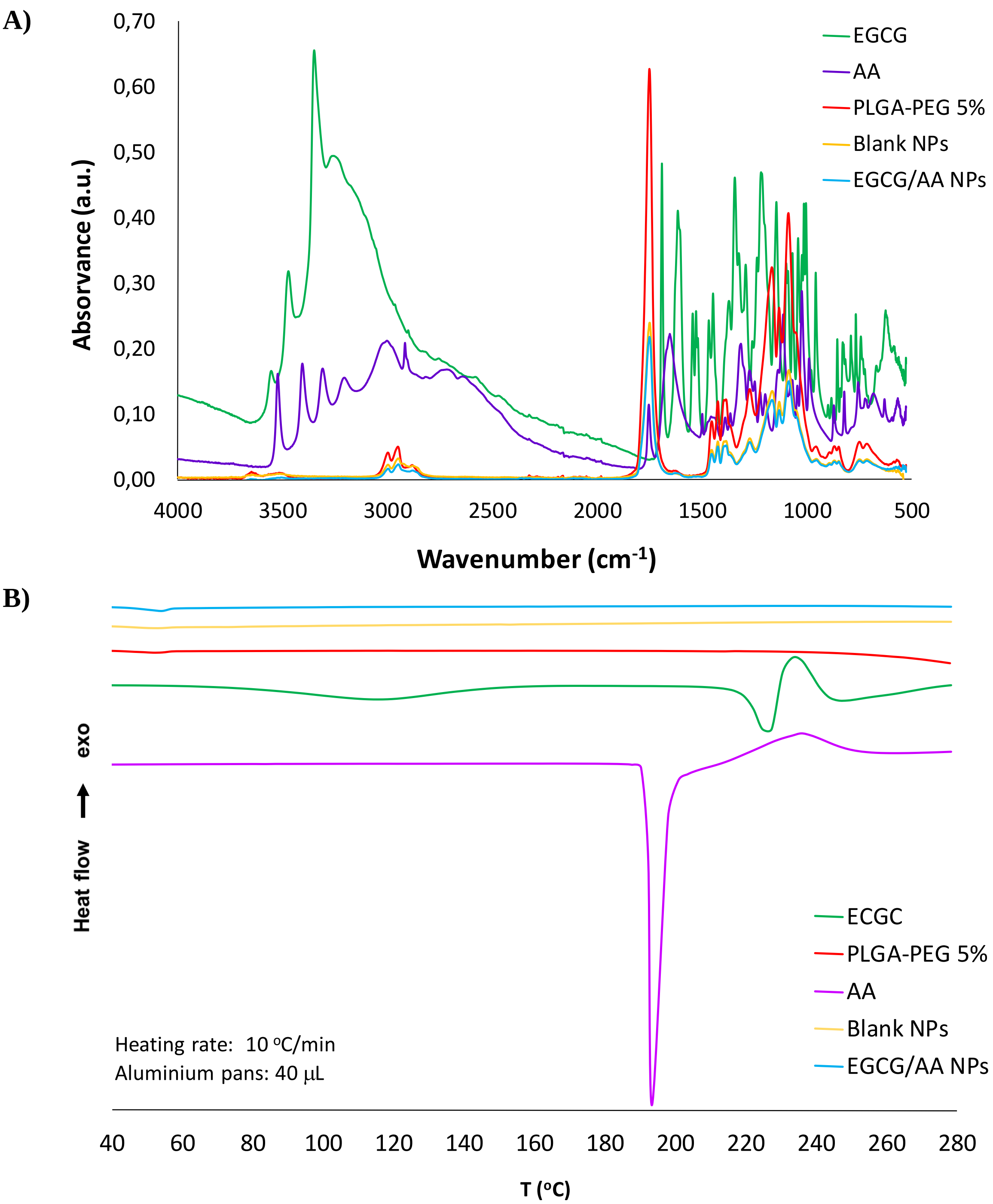

Figure S3. Interaction studies of EGCG/AA NPs compounds**. A)** FTIR spectra. **B)** DSC thermograms.

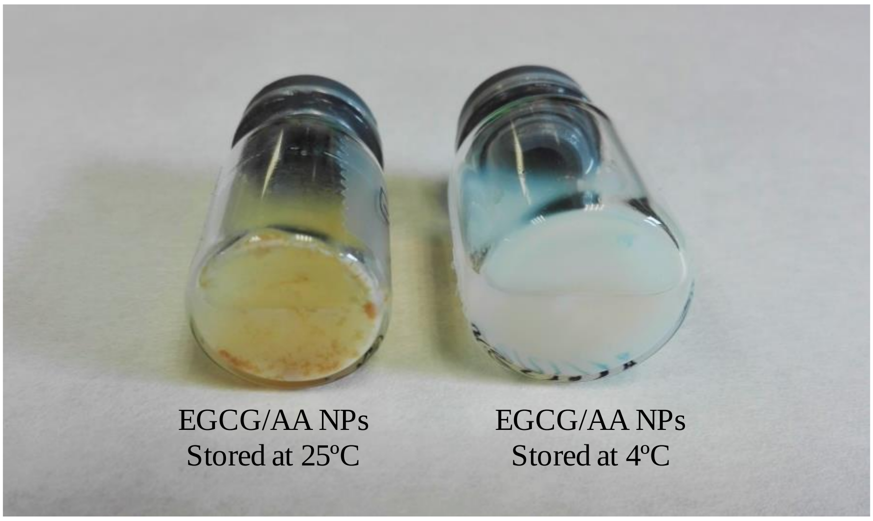

Figure S4. EGCG/AA NPs appearance at 4th month of storage.

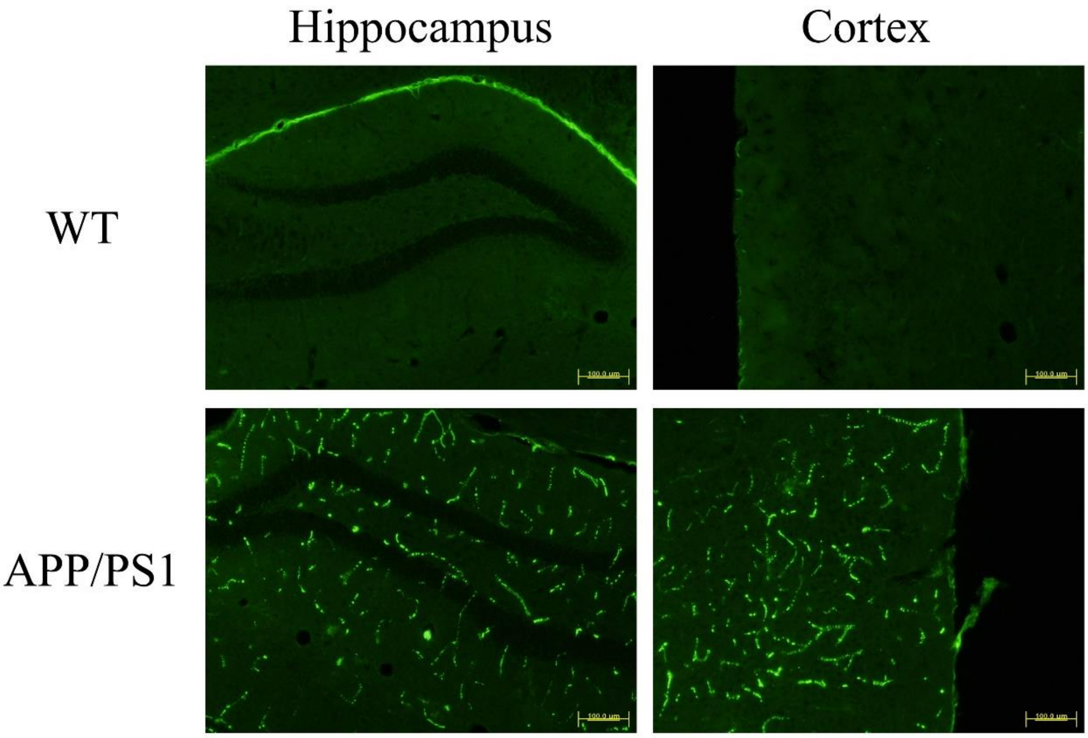

Figure S5. **Endogenous IgG leakage in mouse brains**. C57BL/6 WT and APP/PS1 mice were sacrificed by i.p. injection of ketamine/xylazine (100/10 mg/kg, respectively) and perfused with PFA 4%. Brain coronal sections of 20 µm of thickness were stained with Goat Anti-Mouse IgG (H+L) antibody, AlexaFluor 488 Green (1:1000; Life Technologies, Cambridge, UK). Image acquisition was carried out with an epifluorescence microscope (BX41, Olympus, Germany).

Tables

Table S1. Physicochemical characteristics of EGCG/AA NPs stored at different temperatures

	**Stored temperature 25ºC**			**Stored temperature 4ºC**		
**Month**	**Size (nm)**	**PDI**	**ZP (mV)**	**Size (nm)**	**PDI**	**ZP (mV)**
**0**	120.3 ± 0.7	0.063 ± 0.012	-12.0 ± 2.1	118.6 ± 1.5	0.072 ± 0.004	-13.5 ± 1.3
**1**	123.2 ± 1.5	0.059 ± 0.020	-10.0 ± 0.8	119.2 ± 0.9	0.082 ± 0.018	-12.3 ± 0.7
**2**	120.9 ± 1.2	0.043 ± 0.008	-12.2 ± 2.8	117.9 ± 0.6	0.062 ± 0.017	-13.1 ± 0.5
**3**	130.5 ± 0.4	0.167 ± 0.018	-12.6 ± 0.8	119.2 ± 1.8	0.093 ± 0.015	-12.1 ± 1.1
**4**	941.2 ± 9.4	0.700 ± 0.282	-	124.9 ± 0.5	0.107 ± 0.011	-12.6 ± 0.2
**5**	-	-	-	128.5 ± 2.2	0.095 ± 0.019	-11.5 ± 1.2
**7**	-	-	-	125.4 ± 2.3	0.121 ± 0.024	-10.3 ± 1.3
**9**	-	-	-	126.2 ± 0.5	0.150 ± 0.030	-10.4 ± 1.5
**11**	-	-	-	90.6 ± 2.5	0.250 ± 0.204	-8.6 ± 0.7

Table S2. ANOVA analysis results of the Aβ plaques depositions and Aβ $_{(1–42)}$ peptide levels

Test	Condition	Tukey's multiple comparisons test	Mean Diff.	95% CI of diff.	Significant?	Summary	Adjusted P Value
**Aβ plaques**	Hippocampal region	APP/PS1 vs. APP/PS1 free EGCG	3.528	0.6070 to 6.449	Yes	*	0.0155
		APP/PS1 vs. APP/PS1EGCG/AA NPs	7.639	4.718 to 10.56	Yes	****	< 0.0001
		APP/PS1 free EGCG vs. APP/PS1 EGCG/AA NPs	4.111	0.9887 to 7.234	Yes	**	0.0081
	Cortical region	APP/PS1 vs. APP/PS1 free EGCG	7.447	4.771 to 10.12	Yes	****	< 0.0001
		APP/PS1 vs. APP/PS1 EGCG/AA NPs	9.114	6.438 to 11.79	Yes	****	< 0.0001
		APP/PS1 free EGCG vs. APP/PS1 EGCG/AA NPs	1.667	-0.9501 to 4.283	No	ns	0.2751
**Aβ $_{(1–42)}$ peptide levels**	Soluble Aβ $_{(1–42)}$ peptide	APP/PS1 vs. APP/PS1 free EGCG	-1.799	-16.08 to 12.48	No	ns	0.9219
		APP/PS1 vs. APP/PS1 EGCG/AA NPs	20.45	6.177 to 34.73	Yes	*	0.0109
		APP/PS1 free EGCG vs. APP/PS1 EGCG/AA NPs	22.25	7.976 to 36.53	Yes	**	0.0073
	Insoluble Aβ $_{(1–42)}$ peptide	APP/PS1 vs. APP/PS1 free EGCG	25.40	-14.96 to 65.76	No	ns	0.2105
		APP/PS1 vs. APP/PS1 EGCG/AA NPs	94.53	54.17 to 134.9	Yes	***	0.0009
		APP/PS1 free EGCG vs. APP/PS1 EGCG/AA NPs	69.13	28.77 to 109.5	Yes	**	0.0046

Table S3. ANOVA analysis results of the behavioural test

Test	Condition	Tukey's multiple comparisons test	Mean Diff.	95% CI of diff.	Significant?	Summary	Adjusted P Value
**MWM**	Escape latency - Learning process	WT vs. APP/PS1	-33.48	-46.76 to -20.19	Yes	****	< 0.0001
		WT vs. APP/PS1 free EGCG	-18.59	-32.27 to -4.902	Yes	**	0.0054
		WT vs. APP/PS1 EGCG/AA NPs	-9.915	-24.81 to 4.981	No	ns	0.2786
		APP/PS1 vs. APP/PS1 free EGCG	14.89	2.159 to 27.62	Yes	*	0.018
		APP/PS1 vs. APP/PS1 EGCG/AA NPs	23.56	9.540 to 37.59	Yes	***	0.0006
		APP/PS1 free EGCG vs. APP/PS1 EGCG/AA NPs	8.673	-5.731 to 23.08	No	ns	0.3615
	Escape latency - Test day	WT vs. APP/PS1	-43.03	-54.50 to -31.55	Yes	****	< 0.0001
		WT vs. APP/PS1 free EGCG	-15.61	-27.78 to -3.442	Yes	*	0.0103
		WT vs. APP/PS1 EGCG/AA NPs	-2.376	-13.85 to 9.095	No	ns	0.9314
		APP/PS1 vs. APP/PS1 free EGCG	27.42	15.25 to 39.58	Yes	****	< 0.0001
		APP/PS1 vs. APP/PS1 EGCG/AA NPs	40.65	29.18 to 52.12	Yes	****	< 0.0001
		APP/PS1 free EGCG vs. APP/PS1 EGCG/AA NPs	13.23	1.066 to 25.40	Yes	*	0.0309

	Time in target quadrant – Test day	WT vs. APP/PS1	10.36	4.360 to 16.37	Yes	***	0.0003
		WT vs. APP/PS1 free EGCG	3.748	-1.939 to 9.435	No	ns	0.2947
		WT vs. APP/PS1 EGCG/AA NPs	3.000	-2.573 to 8.572	No	ns	0.4685
		APP/PS1 vs. APP/PS1 free EGCG	-6.615	-12.05 to -1.177	Yes	*	0.0126
		APP/PS1 vs. APP/PS1 EGCG/AA NPs	-7.363	-12.68 to -2.046	Yes	**	0.0040
		APP/PS1 free EGCG vs. APP/PS1 EGCG/AA NPs	-0.7487	-5.707 to 4.209	No	ns	0.9759
	Time in border – Test day	WT vs. APP/PS1	-35.14	-49.66 to -20.61	Yes	****	< 0.0001
		WT vs. APP/PS1 free EGCG	-7.718	-22.25 to 6.811	No	ns	0.4634
		WT vs. APP/PS1 EGCG/AA NPs	-6.417	-20.95 to 8.113	No	ns	0.6121
		APP/PS1 vs. APP/PS1 free EGCG	27.42	12.89 to 41.95	Yes	***	0.0002
		APP/PS1 vs. APP/PS1 EGCG/AA NPs	28.72	14.19 to 43.25	Yes	***	0.0001
		APP/PS1 free EGCG vs. APP/PS1 EGCG/AA NPs	1.302	-13.23 to 15.83	No	ns	0.9943
**NOR**	Exploration time	WT vs. APP/PS1	28.23	18.03 to 38.43	Yes	****	< 0.0001
		WT vs. APP/PS1 free EGCG	16.76	5.354 to 28.16	Yes	**	0.0023
		WT vs. APP/PS1 EGCG/AA NPs	2.544	-8.122 to 13.21	No	ns	0.9131
		APP/PS1 vs. APP/PS1 free EGCG	-11.48	-21.67 to -1.278	Yes	*	0.0231
		APP/PS1 vs. APP/PS1 EGCG/AA NPs	-25.69	-35.06 to -16.32	Yes	****	< 0.0001
		APP/PS1 free EGCG vs. APP/PS1 EGCG/AA NPs	-14.21	-24.88 to -3.547	Yes	**	0.0059
